# How Physical Information Underlies Causation and the Emergence of Systems at all Biological Levels

**DOI:** 10.1007/s10441-025-09495-3

**Published:** 2025-03-25

**Authors:** Keith D. Farnsworth

**Affiliations:** School of Biological Sciences, Queen’s University Belfast, 19 Chlorine Gardens, BT95DL Belfast, UK

**Keywords:** Downward causation, Emergence, Synergistic information, Ecological structure, Positional information

## Abstract

To bring clarity, the term ‘information’ is resolved into three distinct meanings: physical pattern, statistical relations and knowledge about things. In parallel, three kinds of ’causation’ are resolved: the action of physical force constrained by physical pattern (efficient cause), cybernetic (formal cause) and statistical inference. Cybernetic causation is an expression of fundamental (necessary) logical relations, statistical inference is phenomenological, but physical information and causation are proposed as what actually happens in the physical world. Examples of the latter are given to illustrate the underlying material dynamics in a range of biological systems from the appearance of ‘synergistic information’ among multiple variables (mainly in neuroscience); positional information in multicellular development; and the organisational structure of ecological communities, especially incorporating niche construction theory. A rigorous treatment of multi-level causation is provided as well as an explanation of the causal power of non-physical information structure, especially of interaction networks. The focus on physical information as *particular pattern*, echoing the insights of Howard Pattee, provides a more physically grounded view of emergence, downward causation and the concept of ‘closure to efficient causation’, all now prevalent in the organisational approach to biology.

## Introduction

### Disambiguating information language

The term ‘information’ is one of the most overworked in science: one word used to represent so many different meanings that it may have become an impediment to progress, especially in fields such as neuroscience and systems biology where it is most needed. Well known, but sometimes overlooked, is the fact that Shannon’s ‘information’ theory is really a theory of the *statistics* of data communications, though it has proved tremendously adaptable and expandable as the basis of metrics of increasing sophistication, such as the recently introduced integrated information decomposition (*Φ* ID) measures of association in multivariate data (Luppi et al. 2021; Mediano et al. 2022, 2019). Shannon’s information (the complement of entropy, here termed statistical information * I*_*s*_) is famously not about ‘meaning’, but less frequently acknowledged, it is also not a thing in itself, but rather is a quantification of a statistical relationship among patterns in data: * I*_*s*_* I*_*s*_ is a metric. The patterns it describes, if *material*, are physically embodied as the distribution of matter or energy in spacetime and in some fields relevant to biology these patterns are also referred to as ‘information’; here termed physical information $I_p$. The complexity of physically embodied patterns can be quantified using another kind of statistical information metric, following Chaitin (1966); Kolmogorov (1965) and later developments, such as ‘Algorithmic Information Content’ (Chaitin 1990), which literally counts the number of differences in the pattern, discounting repeats (i.e. maximally compressed * I*_*s*_), exemplified with biological applications (e.g. Jiang and Xu 2010; Menconi 2005).

Physically embodied pattern provides a set of constraints on the degrees of freedom of a physical system by specifying the relative coordinates of its parts (e.g. atoms). These constraints (boundary and initial conditions that particularise a physical system (Polanyi 1968)) establish the physical form of the system and the form instantiates the information that is the set of coordinates of its parts. $I_p$ is the particularisation of relative coordinates from among all possible for the system, the complement (or converse) of Shannon entropy, embodied in the *form* of the system (see Farnsworth (2022), for more detailed explanation). Examples of embodied information include nucleotide sequences and the 3D pattern of electrical charge on a molecular surface, or the 2D pattern of light flux on a retina; these are all physical things, not metrics. Taking the last example, it would be normal to refer to such a pattern on the retina as conveying information about the scene beheld by the eye. In this sense the term ‘information’ entails being what philosopher Daniel Dennett called *intentional*—i.e. about something—implying that the receiver (in the Shannon sense) is something for which the information can be significantly informative, i.e. it could have meaning for the system. We can refer to this as semantic information $I_m$ and consider it a precursor to meaning (i.e. having potential for meaning, given an appropriate context). It is semantic in the sense that it could bear on a decision that the receiver system may make (evoking MacKay ’s 1969 idea popularised by Bateson (2015) as “a difference that makes a difference”). The intentional character (*sensu* Dennett (1991)) of $I_m$ may be interpreted as a sign, in which case the information is a sign vehicle (Peirce 1998), but more generally, $I_m$ can be defined as potentially interpreted information, implying the need for an interpreting system and placing $I_m$ internal to that system where it is a representation of some external information $I_p$. Meaning in this sense does not imply thought; for example, Adami (2015) investigated the probability of $I_m$ spontaneously arising from $I_p$, referring to $I_m$ as information and $I_p$ as entropy in assemblies of hypothetical molecular patterns. There, the meaning was to cause an action that contributed to self-replication, hence the emergence of ‘life’ (i.e. for Adami (2015), information was not the sequence, it was the meaning of the sequence). Generalising beyond the mind (where $I_m$ is consistent with the idea of knowledge about things), to include all systems capable of taking a specific action upon encountering specific information, then $I_m$ is information internalised within a system as $I_p$ for which it has *function* as a representation of some other, exogenous $I_p$.

As an aside, note that Wang (2022) strongly asserts that information cannot be physical (contradicting Landauer (1992, 1996)), but with the distinctions provided here, their argument tuns out to be that $I_m \not \equiv I_p$, which is self-evidently true by definition. Landauer’s idea of information was a constraint on the degrees of freedom in a system (originally a Szilard engine), which is nothing to do with meaning. But $I_m$ is a special kind of $I_p$. It is a) endogenous because it has been embodied as part of a (particular kind of) system and b) it has non-zero mutual information (a kind of * I*_*s*_) with some other $I_p$ that is not a part of the system (i.e. exogenous $I_p$). The particular kind of system needed for endogenous $I_p$ to become $I_m$ is one which is able to interpret the exogenous information using the endogenous, and this interpretation gives the exogenous information its meaning. Meaning^1^ is therefore necessarily relational: $I_m$ is (truthfully—Floridi (2005)) *about* something, so it should not be surprising that the existence of mutual information is a characterising feature of any meaningful information, nor that meaning requires the formation of a relation between the endogenous and exogenous information (see Corominas-Murtra et al. (2014), for an engineering application of relation in this context). The most elementary relation is that of comparison: for example in comparing the current value of an exogenous signal with its associated homeostatic set point to inform the system of the internal adjustments currently necessary—this being also the most elementary form of goal directedness (Farnsworth 2017; Heylighen 2022). Homeostasis is therefore the elemental property of a system for which information can be *about* something: a perception of the environment (internal as well as external) is compared to internally embodied $I_p$—in this case, the homeostatic set point. In its most elemental form, the comparison is reduced to a binary determination (exceeds, or does not exceed, the set point), in which case the element of information—the binary bit: one /zero (or more fundamentally, presence / absence)—is transformed to the element of meaning: true / false,^2^ by the elemental form of interpretation that is logical comparison. In general, interpretation means some combination (through processing) of exogenous with endogenous information by a system to produce resultant information which can affect the next state or behaviour of that system (as described in Corominas-Murtra et al. (2014)).

Finally, although information is always embodied as $I_p$, it can be useful to imagine the information hypothetically separate from its medium of embodiment as a formal statement that is realisable in multiple media without specifying any particular medium: this idea of information will be referred to as formal information $I_f$ and it is the commodity of cybernetic systems (hypothetical systems of disembodied information). $I_f$ is useful for understanding computation and related processes, but it is only an intellectual tool, since physical information is always embodied as a configuration of matter or energy.

### Disambiguating causation language

**Fig. 1:**
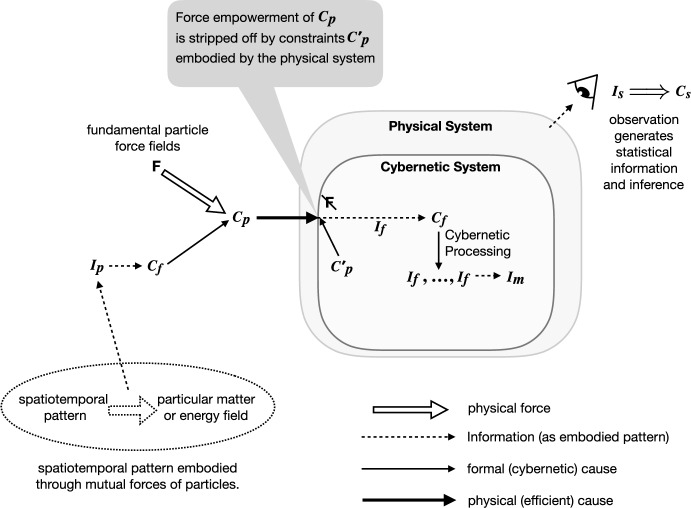
Different kinds of information and causation are explained in context with this diagram. From the lower left, physical information $I_p$ is created by arranging particles or energy fields into patterns and this $I_p$ can act as the formal cause $C_f$, which when empowered by the physical forces emanating from the particles that it arranges, becomes physical (efficient) cause $C_p$. The transducer and cybernetic processing system are themselves information embodied by the physical system. The transducer produces efficient cause $C'_p$ which strips $C_p$ of its force empowerment, leaving just the information embodied as $I_p$, which can then perform constraint within a cybernetic system, where it is described as $I_f$. Cybernetic causes $C_f$ combine it with other instances of $I_f$ and perform processing in the cybernetic domain (logic), for example to create an interpretation, giving rise to meaningful information $I_m$. External to this, an observer (having another cybernetic system) may gather information from this system’s behaviour, inputs, etc., as $I_p$, which can be processed by the observer to produce statistical information * I*_*s*_ and from it infer empirical causes $C_s$

The idea of information affecting the state of a physical system (implying causation by information) remains contentious, but the controversy probably arises from ambiguity in the word ‘cause’. Alongside the three meanings of information, we find three distinct kinds of causation (Fig. 1): (a) statistically inferred cause $C_s$ (observed regularities quantified by statistical information) as in Pearl (2009) (and founded on Hume’s empiricism); (b) cybernetic—logical necessity—causation $C_f$, which is aligned with Aristotle’s formal cause and c) physical causation $C_p$ (Aristotle’s efficient cause), which is constrained action of physical force (Farnsworth 2021, 2022). There is, of course, a connection among them: the constraints applying to physical force to produce $C_p$ consist of $I_p$ and when this embodied information is considered in disembodied abstract (as formal cause), the (hypothetical) processes resulting from interactions among its parts constitute $C_f$, i.e. a set of disembodied logical relations that can be organised as a cybernetic system. A cybernetic system, then, is the hypothetical result of isolating a network of logical relations from its physical embodiment; it propagates the logical consequences of a given $I_p$, strictly excluding any effect of physical force. For that to be embodied in physical form, it must be arranged so that the (unavoidable) effect of physical forces within the system are constrained to act solely as the dynamic substrate for propagating the logic (e.g. driving electrical current in electronic computers, or similarly, ion fluxes in biological systems such as neural networks, or the electrodynamic forces that cause conformational changes in biological molecules). The particular arrangement that constrains the inevitable forces to act solely to make the cybernetic logic proceed dynamically, is itself physical information, in this case setting the coordinates of all the parts constituting the physical embodiment of the cybernetic system (for example the geometric arrangement of logic gates on a silicon chip, or the topology of biochemical signalling pathways). The $I_p$ of this organising constraint is often overlooked, especially in studies of cybernetic systems, but is essential in explaining the physical basis of all informational systems, including life (provides more detail Farnsworth (2022)).

Lastly, statistically inferred causes ($C_s$) are empirical relations among observable embodied patterns, interacting according to the formal (cybernetic) regulations $C_f$. Since we can observe relations among purely cybernetic variables as well as among physical variables, $C_s$ can arise from either, but this does not imply that they are the same. As a product of observation, $C_s$ is dependent upon an observer and therefore on the undeclared organisational information of the observational system, where it can be found as an instance of $I_m$ (i.e. consider the observer as an additional physically implemented cybernetic system in Fig. 1, having the properties necessary for $I_m$ to exist). Semantic information (about things) is now revealed to be a special case of $I_p$ in which $C_s$ is inferred from $I_f$. When a pattern $I_f$, considered internal to a system *S*, forms such that it shares mutual information with some external pattern *E*, we conclude that *S* has information $I_m$ about *E*. Then $I_m$ can be called functional if it informs *S* on how to act—in the most elementary case by adjusting some internal quantity in the direction needed for homeostasis and more generally, in a way consistent with the concept of free energy minimisation in the context of active inference (Friston et al. 2013). Organisms act ($C_p$) upon perceived exogenous information by representing it through inferred causation $C_s$, seeking to minimise the error in their anticipation of it through cybernetic processing $C_f$.

The most obvious biological examples of patterns that embody information which may be interpreted (Pattee 1969, 2001; Deacon 2021) are information oligomers (RNA and DNA) and polypeptides (as linear patterns) and the proteins they produce, which consist of three dimensional patterns, typically described by primary, secondary and tertiary structure. When a set of proteins are assembled into a nano-machine such as the ATP synthase complex, the higher order pattern of their relative locations and orientations embodies information at that higher organisational level: a pattern of patterns. When this hierarchy is formalised as distinct levels of organisation, it is possible to examine the causal relations among levels to address questions about e.g. downward causation (Jaeger and Calkins 2012; Ellis 2012; Bechtel 2017; Farnsworth et al. 2017b) and the ‘closure to efficient causation’ (Rosen 1985a) used to explain autopoiesis (Bich et al. 2016; Mossio et al. 2013) and reinterpreted for the cell by Hofmeyr (2021). The premise here is that these matters, fundamental to understanding biological systems, are best explained in terms of explicitly physical information and causation, following (Farnsworth et al. 2013, 2017a; Farnsworth 2018, 2021, 2022). The aim, therefore, is to isolate physical information, distinct from any other kind and to show how it forms the basis of causation, including upward and downward causation in a variety of biological systems: developmental, neurological and ecological.

## Synergistic and Integrated information

We start with a basic, but conceptually rich phenomenon that has recently attracted much interest, especially among neuroscientists and although it is not physical, its study unveils important physical underpinnings. Cybernetic models of biological systems (sets of formal information $\{ I_f \}$) are often studied using information metrics, including those of relations among multiple signals: these are the * I*_*s*_ metrics based on mutual information and its decomposition. Along with redundant and unique information, synergistic information (SI) is a component part of mutual information found using Partial Information Decomposition (PID) that originated from Williams and Beer (2010) and developed into a sophisticated analysis of multivariate data (see e.g. Gutknecht et al. (2021),for a clear explanation). SI is the information which is only conveyed by multiple ($\geqslant 2$) signals taken together jointly, not because they each carry unique information, but because they jointly carry latent information that must be decoded in some specific way to obtain. At face value, SI appears to produce something out of nothing: information among data channels that cannot be found within them individually. The apparent paradox might be dismissed by noting that SI is not really information at all, but that would overlook the important fact that there really is additional information in a system with non-zero SI. This additional information is implicit, rather than spontaneously appearing; it is embodied at a level of organisation above that of the signals and so is only apparent when observations are taken at the higher organisational level. The appearance of information embodied at a higher level of organisation will be a common theme throughout this work and it is responsible for all cases where there appears to be “more than the sum of the parts”.

As a component of mutual information, which in turn is a calculated statistical quantity describing relational properties of (usually) cybernetic information, obviously SI is a kind of * I*_*s*_. We find something similar in the Integrated Information Theory (IIT) measure $\phi$, that was explained by its authors as “*the amount of information generated by a complex of elements, above and beyond the information generated by its parts*” (Tononi 2008). This $\phi$ is a measure of (implicit) information embodied at the higher level of organisation, derived from the metric ‘effective information’ (Tononi and Sporns 2003; Tononi 2004), which is a measure of all the possible cybernetic effects one hypothetical (bisection) partition of a system’s states can have on the states of the complementary partition. A core component of IIT is its definition of ‘mechanism’ as “*any system element or combination of elements having irreducible cause-effect power within the system*”, i.e. its state constrains past and future states of the whole system whereas that of its component parts do not (hence, irreducibility). This irreducibility implies that a mechanism must entail non-zero SI: specifically the information that is embodied by the mechanism and not by its component parts, in other words, by the organisational structure of the mechanism.

In PID, IIT and much of the multivariate / network theory especially applied to neuroscience, the systems studied are cybernetic models of (real or hypothetical) physical systems. As such, their causal structure is purely composed of $C_f$ and the information considered is formal information and it is quantified by a battery of statistical information metrics all belonging to * I*_*s*_. But underlying all this, in any physical system, formal information $I_f$ is embodied as the spatiotemporal patterns $I_p$ which constrain physical causal relations. The interpretation of $\phi$, and its whole system extension $\Phi$, for physical systems such as brains and networks of logic gates as “*intrinsic information over and above that embodied by their components*” (Albantakis and Tononi 2015), only explicitly refers to the cybernetic organisation (the network topology), allowing us to overlook the physical structure of these systems which is set in advance of the IIT analysis. At least some of the information ‘over and above that embodied by the components’ is to be found in that *given* structure. This is clearest in the case of cellular automata for which interactions are topologically restricted to neighbouring cells, implying spatial structure (embodied $I_f$) at a level above that of the automaton cells. Similarly, the circuitry of interconnection among logic gates and of neurons in a brain is set by a network topology in formal (cybernetic) space to (merely) imply a physical pattern $I_p$ at the higher organisational level. The information measured by $\phi$ is what can be completely separated from that specifying the behavioural (cause and effect) repertoires of the components. It must therefore be found in the connections among those components, including the limits in physical space of their interactions (e.g. a restriction to physically neighbouring cells in a cellular automaton). Even when there are no physically predetermined links, e.g. for organisms in an ecological community or for cells in a tissue, the system-level information that is not embodied by the parts, must be embodied by system-level pattern constraining the distribution of their interactions. In biological systems, this pattern usually emerges from self-organisation to produce a persistent (self-reinforcing) spatiotemporal distribution of organisms (in ecological community structure) or cells (in organism development), both cases to be examined in later sections.

### Examples of SI revealing the underlying physical systems

To understand underlying higher level structure more precisely, we will take a simple, but revealing example. Consider a single source of information, detected by two spatially separated receivers so each receives identical information, but one is time-delayed relative to the other, e.g. two radio antennae set apart, listening to a distant transmitter. Labelling the signal from the source as $s_0$ and from each antenna as $s_1$ and $s_2$, we can examine the mutual information among them. In PID, the two received signals are identical, so obviously they cannot have any unique (*U*) mutual information, hence we say $U_1 = U_2 =0$ and being identical, the signals are completely mutually redundant (*R*), i.e. $R_1=R_2~ =M(s_1;s_0)=M(s_2;s_0)$, (*M* for mutual information). In general, not all the mutual information among the signals $s_1$ and $s_2$ is accounted for by redundant information: synergistic mutual information $S \ne 0$. Given we have two identical copies of the original information, the only possible source of this SI is the time-delay in arrival of one relative to the other. That time delay is a physical spatiotemporal difference, embodied by, and expressing, information at an organisational level above that of the antennae. Since there is a single source of the information (the transmitter), the source of SI must literally be the information embodied by the physical separation of the two antennae and the transmitter. To emphasise: the SI is entirely and only associated with the geometric configuration of source and receivers (this is information embodied at a level of organisation above that of the transmitter, receiving antennae and the information they carry). In practice, since the positions of the receiving antennae are known, this geometric, configuration informing, SI tells the observer the direction in which the transmitter lies (e.g. radio direction finding). Stereo hearing and binocular vision are elaborations of this example, but require far more processing of the signals than the simple subtraction used in radio direction finding. Their need for more complicated (hence information rich) processing (i.e. decoding) suggests further opportunity for SI to arise.

**Fig. 2:**
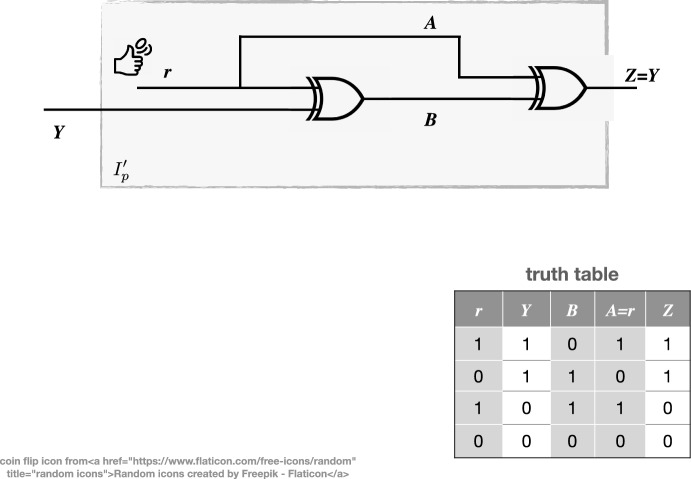
The XOR logic gate produces purely synergistic information in signals *A* and *B* from a binary input *Y*, combined with a random signal *r*. Nothing of *Y* can be discerned from *A* or *B* alone, making for an effectively simple cypher system. When *A* and *B* are combined using another XOR gate, the product is *Y*. Synergistic information is not produced from nothing: in this case the information $I^{\prime }_p$ embodied as a particular configuration of matter, e.g. a silicon wafer, in the form of the XOR gates and associated wiring (grey shaded box), is responsible for its production

A classic pedagogical example of SI (e.g. Gutknecht et al. 2021) imagines a pair of random Boolean source signals (*A* and *B*) which are then combined by the Boolean ‘exclusive or’ (XOR) operation to give a receiver signal *Z* (Fig 2). Signals *A* and *B*, being random, consist of entirely unique information and there is zero redundant information, so we may expect $M(A,B)=0$, but here it is not, because $S(A,B) \ne 0$. The ‘trick’ is that though the signals are random, they are not independently so, because we have *implicitly* determined they are related by XOR. This relation would not be obvious to an observer of the two signals A and B unless they knew to combine them with XOR: in effect the relation is latent organisational information at a level above that of the signals. PID holds that in general, the information *Z*, hidden among *A* and *B* and quantified by SI, is accessible only when both signals are observed in *combination*, but it misses stating what specific kind of combination is required. To explain the specificity of the implicit information that makes SI nonzero in this example, imagine a source signal *Y* (Fig. 2) which is logically combined with a random signal *r* by XOR, then if *A* is the signal *r* and *B* is the combination, it is clear that *A* and *B* are related to one another by XOR. Indeed, the whole system can act as a sort of code for transmitting *Y* to the receiver as *Z*, which combines *A* and *B* using another XOR to recover $Z=Y$. In general, a code is a transformation of information from one domain to another (see Farnsworth (2023), for discussion of domain transformations) and in this case XOR is the code (more precisely termed a cypher in this case). An observer of signals A and B would see them as random unless they processed them through combination with an XOR-gate. If they did that, they would record a maximal SI, and from that could deduce the existence of a particular organisation (a spatiotemporal pattern $I_p$) of the physical system producing *A* and *B* that included the effect of an XOR-gate (the upstream gate combining *Y* and *r*). this upstream gate and its associated wiring is the latent, unobserved, physical structure that is logic ($I_f$) embodied as physical information $I^{\prime }_p$ at an organisational level above that of the signals. This pattern can be deduced from *A* and *B*, using the particular apparatus of another XOR-gate because it has been transformed from the information domain of $I^{\prime }_p$ to that of the signals, by the $I^{\prime }_p$ structure acting as a cypher.

Transformations of physical information from one domain of embodiment to another are defining features of living systems. As well as being enacted by transducers, such as transmembrane ligand receptors, they include transformations from one organisational level of embodiment to another, enabling inter-level effects (Sect. 3). Such effects are of special interest in neuroscience because they form the basis for raising inter-neuronal processes to the level of integrated functions of perception, cognition and, according to most theories of mind, ultimately to consciousness, which is the focus of IIT.

IIT conceptually inverts Pearl ’s 2009 derivation of causal structure from mutual information by intervention, to derive information structure from revealed causal associations. There is a strong conceptual relation between $\phi$ and SI, exemplified by the ‘whole minus sum’ $\Phi$ of Mediano et al. (2022). But the combination of these two approaches (as $\Phi$ID) is normally applied to ‘information flow’ (meaning change from current to future or past to current states), in which information can generally move among the PID information ‘atoms’ when stepping forward in time: hence the use of a lattice of transfers from every kind of atom to every other, over the time step (illustrated in Fig. 1 of Luppi et al. (2021)). This in turn implies the movement of information from one organisational level to another (e.g. from synergistic to unique), requiring not only the copying of it from one to the other, but also its deletion from the source atom, in one logical time step. That is not a problem in principle for cybernetic information and clearly not for information metrics, but it could be one for physical information because transferring $I_p$ from one organisational scale to another is a domain transformation (Farnsworth 2023), which is only possible using a suitable transducer, such as an embodied code. Once again, the solution to this problem is found in the latent information embodied by the network of interactions and among the component parts, e.g. in the update rules for cellular automata, which implicitly performs the role of the transducer code. Indeed for the cellular automaton, the (predetermined) dynamic generating rules, including the topological relations among cells, constitute this code. In its operation, information observed at the individual cell level is transferred to that of the whole automaton through the sequence of logical relations among cell states, so the observed time series of states, decomposed as a network of PID atoms, reveals the movement of information from individual cells to the whole. Only when all state transitions have been observed, can the $\Phi$ and related metrics be found: these metrics quantify the $I_f$ that (a) is implicit in the structure of the cybernetic system and equivalently, (b) encodes the implicit information that is ’revealed’ by SI.

## Multi-level control by physical information

The idea of multiple levels in a physical context is that patterns can be formed as a nested hierarchy of patterns of patterns. For a level *L* pattern to exist, it must be a constraint on the relative positions in spacetime of multiple instances of lower level ($L-1$) patterns and therefore the existence of these $L-1$ patterns is a prerequisite. In biology, atoms are the obvious first level pattern: notwithstanding quantum uncertainty, they are spatial organisations (hence constraining information) of subatomic particles and molecules are clearly organisations of atoms (both levels emerging from free energy minimisation). Because physical forces are strictly base-level phenomena (described as the exchange of virtual sub-atomic particles, or forcefield quanta), $I_p$, whatever its level, only ever constrains the action of forces at the base level ($L=1$)^3^ of subatomic particles from which forces emanate. But constraint on the configuration of particles is independent of the organisational or spatiotemporal scale at which the constraining information is embodied (the constraint is only the specification of coordinates, which can be at any scale). Therefore, efficient causation $C_p$ can result from information embodied at any organisational level, constraining forces at the base level. In general, for any level *L* in an organisational hierarchy of pattern, let $^{L}\textbf{A}$ be an assembly of $n_L$ components (lower-level assemblies) $\{^{L-1}\textbf{A}_1 ~\cdots ~ ^{L-1}\textbf{A}_{n_L} \}$, configured in space as a pattern of patterns $^{L}\textbf{Z} = \{^{L-1}\textbf{Z}_1 ~\cdots ~ ^{L-1}\textbf{Z}_{n_L} \}$, where $^{L-1}\textbf{Z}$ are the Euclidean space coordinates of level $L-1$ elements in a level *L* pattern. For the first level assemblies, we have $^{1}\textbf{A} = \{e_1 \cdots e_n \}$, where $e_i$ are fundamental particles, each having an associated force-field $^1\textbf{f}_i$, the vector sum of which $=\,^{\phantom {1}1}\textbf{F}$ (for which we will usually drop the explicit level identifier) and these are configured in space by $^{1}\textbf{Z} = \{\textbf{z}_1 \cdots \textbf{z}_n \}$, where each $\textbf{z}_i$ is the set of relative coordinates of particle $e_i$. Every pattern constrains the action of forces by setting the relative coordinates of particles from which they emanate. Most obviously and conventionally, at level 1, we have $^1 \textbf{Z} ~\triangleright \textbf{F}$ ($\triangleright$ represents ‘constrains’). Associating information embodied at level *L* (i.e. $^L I_p$) with $^L\textbf{Z}$ and associating physical causes found at organisational level *L* (i.e. $^L C_p$) with the $^L\textbf{Z}$ constraints on the action of $\textbf{F}$ (by specifying the location of particles from which the forces emanate) gives for level 1: $^1 I_p ~\triangleright ~ ^1C_p$.

This can immediately be generalised since it is true, irrespective of level: every $^{1}\textbf{Z}$ constrains the action of forces emanating from the particles it places in space and any higher e.g. $^{2}\textbf{Z}$ adds further constraints by virtue of specifying the relative position of each member $^{1}\textbf{A}_i$ within $^{2}\textbf{A}$. For example if $^{3}\textbf{A}$ was a phospholipid molecule and $^{4}\textbf{A}$ a phospholipid membrane, the arrangement of molecules in the membrane would be $^{4}\textbf{Z}$, with associated $^4 I_p$ and any physical causes *specifically* depending on this membrane structure would arise from $^4 I_p ~\triangleright ~ ^4C_p$. Chemically, all the phospholipids have well known amphipathic properties, based on an electrostatic pattern ($^3I_p$), by which they also spontaneously form sheets, giving rise to selectively permeable membranes that can be folded into tubes and enclose spaces in three dimensions as e.g. cell teguments: all these are cases of $^{4}\textbf{A}$ with associated $^4 I_p$. The ‘tube’ and ‘enclosure’ properties are strictly a result of configuration $^{4}\textbf{Z}$ and in the context of a higher biological system level, they *function*, e.g. by directing flow and selectively constraining diffusion. In turn, according to the *causal role* interpretation of function (Cummins 1975; Farnsworth et al. 2017a), these functions are a subset of the property-dependent causes of level 4: $\{^{4}C_p \}$ (recall $\{ \cdot \}$ denotes a set). More precisely (than property-dependent), these functions result from the $^{4}\textbf{Z}$ arrangement’s specific contribution to constraint on the action of $\textbf{F}$ emanating from all the particles constituting the membrane. In general, $^L I_p ~\triangleright ~ \{^LC_p \}$, which compactly expresses efficient cause by same-level constraint.

Several authors (cited below) have argued that causation cannot cross over levels (upward or downward), but by resolving physical causation into forces and the $I_p$-constraints upon their actions, we can see a way for this to happen, supporting the insights of many such as Perovic (2007), Jaeger and Calkins (2012), Ellis (2012), El-Hani and Emmeche (2000) and Ellis (2020).

The main objection to inter-level causation is that it appears to over-account for causes, i.e. causal redundancy arises in contradiction of the *exclusion argument* (especially following Kim (1989)). The idea of downward causation has been the prime target of criticism using this argument, but upward causation is equally ruled out by the exclusion argument (see e.g. Raatikainen 2018) and also by the *causal equivalence principle* (Yurchenko 2023): a deduction from causal set theory (Bombelli et al. 1987). For specifically physical causation, it is true that, due to physical causal closure, an event can have only one sufficient physical cause. However, the physical basis of the exclusion principle applies only to the physical force constituent of causation, leaving physical information able to constrain the forces from multiple levels simultaneously. This is because $I_p$ is nothing more than the determination of the spatiotemporal coordinates of elementary particles, which may be given at multiple organisational levels, whilst forces are strictly confined to the base level. In other words, the causal-closure basis for the exclusion argument (and also causal set theory approaches) refers only to the base level of fundamental process of physical causation at the Plank scale where fundamental particles exchange conserved quantities, resulting in physical forces. If we were to account for multilevel causes by including the physical forces in each alleged cause of an event, then we would indeed be overdetermining the event, but only because we would have mistakenly included the forces more than once. That is what happens when a conventional (unresolved) idea of efficient cause is used in accounts of downward causation. Instead, we should count force as a single unitary component of physical causation, but having done that, we are free to accumulate multiple constraints by physical information embodied at multiple organisational levels ($\cdots , ~^{L-1}I_p, ~^{L}I_p, ~^{L+1}I_p, \cdots$) on the action of the vector sum of forces $\textbf{F}$ emanating from the set of base-level member components $\{e_1 \cdots e_n \}$. Significantly, since cybernetic causes definitively do not involve forces, they are not subject to the exclusion argument or any other objections to trans-level causation, which is why causal talk in IIT, etc. is reasonable (but see e.g. (Romero 2015) for discussion of this in statistical inference).

A kind of downward causation appears immediately from the interpretation of physical cause given above: since forces are confined to the base-level, effective constraints by higher level information will appear as $^{L}I_p ~\triangleright ~ ^1C_p$, ($L>1$). It would be more precise to call this *downward constraint*, rather than downward causation, though the effect is the same. Less pedantic is the possibility of inter-level causation through interaction among multiple levels of $I_p$ constraining the action of $\textbf{F}$ simultaneously: the net effect of which is information at one level constraining information at another. Again there is a rather obvious sense in which this must be the case, since by definition an $(L+1)$ pattern is the constraint of arrangement on level *L* patterns. But the established meaning of downward causation is that $(L+1)$ configuration affects the *state* of level *L* patterns, rather than merely moving them around. This implies that $^{L+1}I_p$ constrains configurations internal to level *L*. To explain how that is possible and to complete the account, we must include the physical implications of $C_p$, i.e. the role of forces undergoing constraint of action (obviously this is neither relevant nor a limitation for cybernetic systems). In a physical system, the locations of level *L* patterns relative to one another can have a substantial effect on their states and even their constitutions as long as they are sensitive to their environment, which includes one another.

Taking a practical example, the biochemical mechanisms of life in a cell, at a molecular level ($^3I_p$), consist of interactions by close neighbours where molecular forces are constrained to enact e.g. cleaving, conformational change and ligation. The coordination of these molecular actions is a case of $^{3+j}C_p$, ($j\geqslant 1$), i.e. constraining the transformations (instances of $^3C_p$) which are only made possible by arranging the relevant molecules close enough and in the right orientation. This selective bringing together of molecular actors is not just random, it is the result of organisational work done by supramolecular processes, explicitly in the case of eukaryotes through compartmentalisation, but still implicitly by timing and the management of numbers of each molecular species in prokaryotes, all under genetic control: i.e. regulation by the whole cell and its organising processes as $^{3+j} I_p ~\triangleright ~ \{^3C_p \}$. Also, molecular scale interactions, taken together, often comprise systems including signalling and control pathways, which feed back to the genetic control system via transcription, translation and related regulation so that lower level information patterns affect the organisation at the higher level of the cell: upward causation $^3 I_p ~\triangleright ~ ^{3+j}C_p$.

It should be emphasised that downward causation requires lower level patterns to be properly a part of the higher level source of causation, hence exogenous organisation (externally imposed pattern, e.g. an environmental gradient in ecology) is not eligible as a source for downward causation. Denoting parthood by $\sqsubset$, from mereology (see van Inwagen 1987), ‘properly a part’ simply means that if *x* is a level *L* and *y* a level $(L+1)$ entity, then . In a nested hierarchy, where $x \sqsubset y \sqsubset z$, it follows that $x \sqsubset z$ (the transivity axiom, for which *z* is a $(L+2)$ pattern). To prohibit exogenous causation, we need to restrict inter-level cause to a single (unbranched) hierarchical chain of parthood relations. That is, if there are two $(L+1)$ patterns $y_1$ and $y_2$, then if $x \sqsubset y_1$, , and vice versa and in general for all levels, so that every pattern can be a part of only one pattern in the level above it. This means that downward causation applies only to exclusive proper parthood (the exclusive referring to prohibition of exogenous control by the rule above). To these abstract conditions, let us add the physical condition that (physical) parthood must imply that the part at least could in principle make a causal difference to the whole. In other words, if a level *L* system were removed from a level $(L+j)$ system of which it is an exclusive proper part, then the state-space of the level $(L+j)$ system would change (note it is for general $j\geqslant 1$). This physical condition means that to be a part of something, there must be a physical consequence to being removed from it, but the condition does not go further in implying that the part must have a function—the consequence may be no more than that the $(L+j)$ pattern is changed by $^{L+j}\textbf{A}$ loosing a member component $^{L}\textbf{A}_i$, with consequent change of embodied information $^{L+j}I_p$ and its constraint on the action of $\textbf{F}$ (recall MacKay and Bateson’s statement that information is a “difference that makes a difference” and the axiom of IIT that if an entity can never have a causal effect, then it (effectively) does not exist (e.g. Albantakis and Tononi 2015). This leads to an interesting conclusion: if removal of a level *L* part has some causal effect on the $(L+j)$ system, then the part has causal effect within the system, hence, $^{L}I_p ~\triangleright ~ ^{L+j} C_p$ and downward causation therefore depends on, at least the possibility of, upward causation. That should not be surprising, since for downward causation, a level $(L+j)$ pattern must exist to affect level *L* patterns and it must not be exogenous to them (else it would not qualify as downward causation), hence it must have been created from them, either by their action or by some spontaneous process (e.g. the self assembly of a virus capsid). Among biological systems, spontaneously formed structure, at levels higher than molecular, is rare compared to construction of structure (e.g. the eukaryotic cell, a tissue or an organ) that arises from its parts arranging themselves following a ‘template’ of formal information that is embodied at the lower level (Hofmeyr (2021) provides a detailed description of the self-construction of the cell as fabrication and assembly guided by $I_f$ embodied at the molecular level). We ought therefore to expect downward causation within biological systems to be coupled with the the upward causation by which $^{L+j}I_p$ is formed through the set of level *L* causes $\{^L C_p\}$, consistent with the general arguments of Noble and followers (e.g. Noble 2012)) about multi-level causation. More specifically, it is a particular combination of upward and downward causation that results in *closure to efficient causation* (Rosen 1985a, b, 1991) in ontological accounts of the cell (Hofmeyr 2021; Vega 2023).

**Fig. 3:**
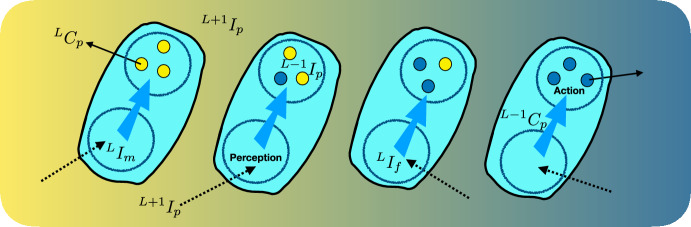
Cells (level *L*) produce (cause $^L C_p$) two hormones (blue and yellow shading) in response to their relative position (positional information $^{L+1} I_p$), thereby creating gradients in hormone level (emergence = upward causation). This in turn is sensed by the cells (perception: $^{L+1} I_p \rightarrow ~^{L} I_f$), which by the internal logic of the cell ($^{L}I_f$), produces molecular level ($L-1$) changes resulting in e.g. cell-differentiation and differential stimulation of hormone production $^{L-1}C_p$ (e.g. as part of homeostatic control)—downward causation. Solid arrows show hormone release to form the $^{L+1} I_p$ pattern; dotted arrows show perception of the local state of this pattern (via a transmembrane transducer effectively transforming $^{L+1} I_p \rightarrow ~^{L} I_f$), which becomes meaningful ($^L I_m$) for the cell because it determines a response (thick blue arrow), actuated by the molecular-level hormone release function ($^{L-1}C_p$), and potentially others. At the tissue level ($L+1$), the assembly of cells $^{L+1} \textbf{A}$ forms a pattern $^{L+1}\textbf{Z}$ to embody $^{L+1} I_p$ at the same level as the hormone gradient $^{L+1} I_p$. Within the cell, $^L I_p ~\triangleright ~ ^{L-1} C_p$, i.e. the (functional) organisational information of the whole cell produces molecular level causes that together form a molecular information processing system that instantiates perception and action.

Indeed, in emergence, level L patterns $\{^{L}I_p\}$ organise themselves into a particular configuration, thereby creating $^{L+j}I_p$, which in turn and along with its member parts, contributes to constraining the action of $\textbf{F}$. In so doing, this $^{L+j}I_p$ may constrain the configuration of level $L-1$ components within each member of $\{^{L}I_p\}$, thereby exercising downward causation. The action of cells (level *L*) in early development of an embryo provides a practical example (Fig. 3). Here $(L+1)$ information is usually embodied as the distribution of concentration in a signal chemical such as auxin in plants (Jeong et al. 2010) and kinases in e.g. planarian regeneration (Umesono et al. 2013) and more complicated signalling systems in embryogenesis (e.g. Yan et al. (2018)). With the recently discovered addition of bioelectrical signals (Oviedo et al. 2010; Levin 2017; Levin and Martyniuk 2018), the “morphogenic problem solving” (organisation of individual cell actions to form a tissue) has been recognised as *computation* (i.e. information processing) in which the tissue level $(L+1)$ shows sufficient cybernetic autonomy to be termed an “agential material” (Levin 2023). Despite that, most examples (see e.g. Manicka et al. (2023)) still take the $(L+1)$ information to be a ‘pre-pattern’ providing positional information, i.e. it is exogenous $(L+1)$ information that cannot qualify for downward causation. The only source of emergent $(L+1)$ pattern, so far identified in development is reaction–diffusion mechanics, which is a spontaneous equilibrium finding process, not computation (Wolpert’s 1969 balancing model, and variants of it have been proposed as viable sources of positional information, but they presently lack examples of biological realisation). Although information processing to produce more elaborate patterns than gradients is quite possible, we do not need to assume it to see both emergence (upward causation) and downward causation at work in gastrulation, or tissue formation (Fig. 3).

## How non-physically embodied information can become causal

So far, we have been thinking of patterns and configurations as distributions of substrate (matter or energy) in physical space with coordinates from which (and distances over which) forces can act. But information is not necessarily so restricted, for example a pattern of connection strengths among neurons in a neural network does not exist in a physical space because the connections and their strengths pay no heed to physical distances and it may even be impossible to transform them into a ‘natural’ physical space where physical distances matter (this is analogous to a ‘Seven Bridges of Königsberg’ type problem in reverse). That is even clearer in the case of an ecological interaction network, which is supra-organism scale formal information having no clear physical embodiment above the organism level. Given this, it seems impossible for such non-physically embodied information to constrain forces and thereby be causal. This section deals with that problem.

Following Farnsworth (2023), all information is embodied in some particular domain consisting of the attributes—medium, basis and scale. The medium is the physical substrate (e.g. electrical fields, carbon concentrations or mechanical forces) in which information is embodied as variation. The basis is the elemental motif of encoding (e.g. the codon or byte) and the scale is the spatiotemporal scale at which it is embodied (e.g. the frequency in radio communications or the molecular scale in genetics). Basis and scale are the two aspects of the *space* in which information can be embodied: for information in general, it is not necessarily a physical space, but it must be a metric space. Physical systems consist of substrate (matter and energy, or more fundamentally a wavefunction) distributed in a physical space, hence $I_f$ can be embodied in a more general category of space than the physical space needed for $I_p$. To be general, we will label the space of information embodiment using $[\textbf{M}]$ for any metric space (e.g. the set of interconnections among neurons or among organisms, together with the metric of their strengths, often described by an interaction matrix) and $[\textbf{z}]$ for the more narrowly defined *n*–dimensional Euclidian space ($\mathbb {R}^n$) which supports Euclidean distances (e.g. Pythagoras’s theorem works for the vector sum of forces). The Euclidean space is important for physical causation (at least at biologically relevant scales) since such causation depends on physical forces which only exist in a physical space which, in turn, conforms to Euclidean space over non-relativistic scales.^4^ Recalling that forces are necessarily confined to the base level of organisation, we identify them with their domain as $^{1}F[\textbf{z}]$. For physical cause, the addition of information constraints by $^{k}I_p[\textbf{z}]$ (where *k* is any level) is necessary and we note that this is also in the Euclidean space. This poses a problem for the putative $(L+1)$ information that specifies a topology, such as an interaction matrix (ecology) or a neural network (neuroscience)—the problem is that it is not embodied in $[\textbf{z}]$, but rather some network topology space, say $[\textbf{w}]$ that is in $[\textbf{M}]$, so it cannot be causal and cannot even constrain, since it exists in a different domain to physical embodiment: it is *domain incompatible*. The only physical solution to this is a transformation of the information’s domain space from $[\textbf{w}]$ to $[\textbf{z}]$ (Farnsworth 2023). Whether any such transformation is possible remains an open question. Taking the parsimonious view that it is not generally possible prohibits non-Euclidean information embodiment from contributing to physical cause: $^{L+1}I[\textbf{w}] \ntriangleright ~^{1}F[\textbf{z}]$, hence $^{L+1}C_p$ cannot be based on non-Euclidean information embodiment.

However, there is an indirect way in which $^{L+1}I[\textbf{w}]$ can potentially exercise causal power over the physical domain. That is by influencing some dynamic process *P*(*t*) which reinforces an $(L+1)$ pattern in physical space. Then, *P*(*t*) acts iteratively (or cyclicly) on an initially random distribution of level *L* parts $^{L+1}R[\textbf{z}]$, to work as a filter, gradually reinforcing a *particular* pattern, hence generating some $^{L+1}I[\textbf{z}]$ de-novo. Consider for example the inter- and intra-species competition among plants, specified as a competition matrix on the metric space $[\textbf{w}]$. It has influence on plant population dynamics, leading to an inter-generational change in spatial distribution among species, instantiated as a set of level *L* components (the plants) configured in physical space as an *emerging* level $(L+1)$ pattern: the vegetation distribution. An initially random distribution of plants will become patchy if interspecies exceeds intraspecies competition (of course it could be a lot more complicated), and patchiness is an $(L+1)$ pattern embodying $^{L+1}I[\textbf{z}]$. Once established, this, now physically embodied, information can contribute to causation. This can be put succinctly as $^{L+1}I[\textbf{w}] \overset{P(t)}{\longrightarrow }~ ^{L+1}I[\textbf{z}]$, from which, $^{L+1}I[\textbf{w}] \triangleright ~^{1}F[\textbf{z}]$ becomes indirectly and *emergently* possible and since the emerged ${L+1}$ pattern affects the outcome of e.g. competition among individual plants, or reinforcement among individual neurons, effectively $^{L+1}I[\textbf{w}] \triangleright ~^{L}I[\textbf{z}]$, i.e. downward causation becomes possible, even across domains. This, though, is on the strict condition that information embodied in a non-Euclidean domain (e.g. network topology) can constrain a physical dynamic process, enabling it to transform $^{L+1}I[\textbf{w}]$ into the physical domain via dynamic emergence. In this case, the process *P*(*t*) is influenced by $^{L+1}I[\textbf{w}]$ through constraints on the dynamics (represented by e.g. competition coefficients or interaction probabilities in differential equations of population dynamics, or probabilities of synapse excitements). Importantly, the constraints it imposes via *P*(*t*) are at the individual level (*L*), where it is effectively a scalar, not a pattern needing a space of embodiment. In that way, $^{L+1}I[\textbf{w}]$ acts through the set $\{^{L}I_p[\textbf{z}] \}$ in the physical domain. Although $^{L+1}I[\textbf{w}]$ is connected to the physical domain through the narrow passage of a scalar quantity, variation in that quantity among members of $\{^{L}I_p[\textbf{z}] \}$ (e.g. growth rates of individual plants) can induce the emergence of $^{L+1}I_p[\textbf{z}]$ via mutual interactions that are determined in physical space (e.g. nearest neighbours). There remains one more condition on this space-transformation process: it must operate at a timescale that can keep up with randomisation of pattern at the $L+1$ scale.

To illustrate, consider a well mixed ecological community, e.g. a gut microbial community in which process chain networks occur along with competition among microbes. This community is unlikely to support the kind of dynamic emergence needed to transform information from $[\textbf{w}]$ to $[\textbf{z}]$. A well mixed $(L+1)$ system is constantly undergoing randomising movements of its level *L* parts, so all $(L+1)$ structure is dispersed as it emerges and there cannot be levels higher than the organism to produce downward causation from $(L+1)$. That is, the $^{L+1}I[\textbf{w}]$ is unable to transform into $^{L+1}I_p[\textbf{z}]$ because the rate of dynamics *P*(*t*) is slower than that of mixing in physical space $R[\textbf{z},t]$. Usually, we would expect *P*(*t*) to follow population dynamic rates (perhaps hours) and $R[\textbf{z},t]$ to follow the mechanical churning rate (perhaps minutes). Since, therefore, no $^{L+1}I_p[\textbf{z}]$ can exist in such systems, the networks of process chain and competition interactions among organisms are embodied at no higher level than the organism level *L* (more precisely, it will be at a lower level $(L-j)$, since it depends on organism differences that are embodied in genes and their expression).

### Neural networks

The physical emergence of higher level organisation as a network topology in neural networks is different because interaction strengths in those are not spatially determined (as they were in the plant community). A popular analogy is with airline networks, where air traffic flow is roughly related to airport city (population) size, rather than proximity among airports. With *k* airports represented as network nodes of population size $\textbf{c}=\{c_1~\cdots ~c_k \}$, we can capture $^{L+1}I[\textbf{w}]$ in a $k \times k$ matrix $\textbf{a}$ of internode flow rates $a_{ij}$ (e.g. flight frequencies). Let e.g. $a_{ij}=\gamma c_i c_j$, with $\gamma$ as a scaling constant. This sets a rule that flight frequency is proportional to the product of airport sizes, but any rule will do. Note, this rule embodies $^{L+1}I[\textbf{w}]$ at the airport level (*L*) because every element of $\textbf{a}$ is determined by only airport size. The rule is formal information ($I_f=~^{L+1}I[\textbf{w}]$) and the question of whether it can be embodied as $^{L+1}I_p[\textbf{z}]$ therefore becomes the question of whether it can *emerge* from an initially uninformed network (e.g. the unitary matrix $\textbf{a}_1$, such that $a_{ij}=1~\forall i,j$), along with a specification of $\textbf{c}$. For this, we need a dynamic pattern reinforcement process, or update rule having the effect: $d a_{ij} (t) / d t = \kappa ( a_{ij} (t) - \gamma c_i c_j )$, with $\kappa$ a scaling constant. This dynamic process will cause the links $a_{ij}(t)$ to tend towards their target values set by the formal information rule $a_{ij}=\gamma c_i c_j$, whereupon the rule will be embodied in the frequency of flights (physical flow rates of aircraft). It is easy to imagine a practical process having this effect: demand for flights would likely be correlated with the product of city sizes and suppliers of flights would likely respond (physically) to this demand. By this, the physical information of city sizes $\{^{L}I_p\}$ is transformed into physical information at the network level $\{^{L+1}I_p\}$ in the form of flight frequencies among cities.

In the more biologically relevant case of learning (acquiring $I_m$) by building neural network patterns in an animal brain, we have *k* neurons, with among them, synaptic connection strengths $\textbf{a}$ and firing rates of individual neurons given by $\textbf{c}$, together with some specific restrictions representing living neuron behaviour (those details are not needed here). It is well known that learning is based on reinforcement by repetition of activating stimuli from the presynaptic axon ending to the postsynaptic dentrite side of the synaptic junction in chemical synapses. A memory is thought to be a pattern of relatively strong synaptic connections among a set of interconnected neurons, developed by a dynamic pattern reinforcement process: long term potentiation. This reinforcement both produces and depends upon $\textbf{c}$, with the eventual result that a particular memory *x* can be represented as the particular $(L+1)$ pattern $\textbf{a}_x$ of connection strengths among *k* neurons. Forming $\textbf{a}_x$ would amount to a transformation of information embodiment from the neurons (at level *L*) to the network (level $L+1$); i.e. emergence. Significantly, the $(L+1$) pattern that emerges is physically embodied in the distribution of supra-molecular level patterns (e.g. receptor concentrations) among synapse junctions (i.e. it is a spatial assembly of synapses that differ in functional properties based on their receptors etc.). Because it is formed and maintained through a dynamic process operating on the network $(L+1)$ scale, it has the character of both emergence and downward causation (the latter, by reinforcing molecular scale patterns). This way, $\textbf{a}_x$, despite being a non-Euclidean pattern $^{L+1}I[\textbf{w}]$, is physically embodied by variation of $(L-j)$ pattern (*j* pointing us to the level of molecular machines and pathways within nerve cells and their synapses). That is, $\textbf{a}_x$ constitutes constraining information (pattern) for $(L-j)$ processes, each of which directly constrains the neuron (the physical embodiment of $^{L}I_p$) to which they belong, hence effectively $^{L+1}I[\textbf{w}] \triangleright ~^{L}I[\textbf{z}]$.

The underlying physical process at $(L-j)$ depends on the density of neurotransmitter vesicles and voltage gated calcium channels in the presynaptic bouton and of postsynaptic receptors on each respective side of the synaptic cleft. Very crudely, neurotransmitters and receptors grow in response to demand which increases with neuron firing rate. The result is that among physically distant (long axon), but network-close (high correlation of firing rates) neurons, the synapse sensitivity grows. Of course in reality it is much more complicated (neuroplasticity includes processes of synaptogenesis and dendritic growth stimulated by neurohormone and other signalling pathways (see e.g. Petzoldt and Sigrist (2014)), as well as synaptic pruning) and the reinforcement rule is more subtle and variable (see e.g. Batool et al. (2019),for an introduction), but the principle is the same. In the airport example, network information is embodied in the domain of flight frequency and in the neural network example it is embodied in the domain of synapse sensitivity. In both cases (and perhaps in general), formal information is transformed from a level *L* physical embodiment to a level $L+1$ physical embodiment by a dynamic pattern reinforcement process.

### Ecological community networks

The organisation of an ecological community (level $L+1$) largely depends on information embodied in the anatomy and physiology producing traits (level $L-1$) of organisms (*L*). This has been represented by trait matrix based foodweb models (Rossberg et al. 2006, 2010; Nagelkerke and Rossberg 2014), where the degree of matching between predator and prey traits determines interaction strengths: a phenomenon observed in experimental systems by e.g. Klecka and Boukal (2013). Trait matching underlies other ecological interaction types, for example, the remarkable structuring of pollinator networks (Olesen et al. 2007) and more obviously, host–parasite relationships.

The topology of the community interaction network $\textbf{a} \equiv ~ ^{L+1}I[\textbf{w}]$ is constantly emerging from the realisation of interactions among level *L* components. This is consistent with the definition of soft emergence (Chalmers 2006): a high level phenomena is weakly emergent when it is (at least in principle) explicable in terms of its lower level domain. If all the organisational information is found in $L-1$, then we could in principle deduce community ecology from knowledge of organism traits and $^{L+1}I[\textbf{z}]$ would not exist—that was the situation in the well mixed community described above. Conversely, if there remains any organisational information at $L+1$, then the community will show at least one phenomenon that is strongly emergent because it cannot be deduced, even in principle, from *L* or $L-1$. Thus the level at which information is embodied determines if higher level phenomena are strongly or weakly emergent. The environmental milieu (e.g. soil conditions on land, or water chemistry in aquatic communities) provides the most obvious material for embodying information at $L+1$. If organisational information at that level is independent of the community (e.g., in the short run, geology or climate determined), then it is not a matter of emergence at all (the case of exogenous $^{L+1}I_p$ described above). We have already seen that plant community dynamics can transform $^{L+1}I[\textbf{w}]$ into $^{L+1}I[\textbf{z}]$ as a patchwork of vegetation community variation in physical space. Plant communities come with a retinue of microbial and animal species performing ecological functions such as predation, symbiosis (e.g. mycorrhizae and pollinators) and parasitism and all these interactions can be included in a widened community interaction network (still of the form $\textbf{a} \equiv ~ ^{L+1}I[\textbf{w}]$ ).

One way this whole $\textbf{a}$ might be transformed into physical space is via *niche construction* (Odling-Smee et al. 1996, 2013): the process by which organisms actively modify their environment to create and maintain their realised ecological niche. At a population dynamic scale, organisms indirectly interact through the modified environment which acts as a filter selecting for only those traits that can succeed within it, hence the organisms persisting eventually include a set of interacting specialists for the manipulated environment (many generalists remain). The joint action of niche manipulating organisms, trait filtering and specialisation are mutually reinforcing and together create an identifiably different community, often with a hard boundary (a consequence of reinforcement processes), such as the grass-heather mosaic found on heathlands: a pattern at the meta-community $(L+2)$ scale. More generally, the self-organised emergence of landscape-scale patterns, via ecological interactions, is well known (reviewed by Rietkerk and van de Koppel (2008), Martinez-Garcia et al. (2022)), especially where Turing-like reaction diffusion processes (short range reinforcement with long range inhibition) are seen (van de Koppel et al. 2005; Liu et al. 2013), but these too emerge as meta-community $(L+2)$ patterns. This suggests that ecological interactions can transform $^{L-1}I_p \rightarrow ~ ^{L+2}I_p$, but that is a scale transformation that seems to miss the organisms $(^{L}I_p)$ and community interaction network ($^{L+1}I_f$).

That conclusion might be a subjective matter of interpretation. Meta-community is not clearly a separate ontological level from community, since it can be described in exactly the same terms: as a set of populations regulating one another through a network of ecological interactions. We may therefore interpret meta-community as the emergent phenomenon of spatiotemporal pattern emerging at the community level, then we could dispense with the idea of $(L+2)$. Further, we may (with caution) think of a set of traits belonging to an organism as indivisible parts of the whole organism: characteristics of a level *L* entity. If we do, then niche construction implies that organisms transfer or copy organising information from themselves ($^{L}I_p$) to the community level of formal information, where it acts to constrain the set of possible interactions among organisms and even excludes some from the community: $^{L}I[\textbf{z}] \triangleright ~^{L+1}I[\textbf{w}]$. But in doing this, indirectly via the reinforcement dynamics (*P*), community level pattern emerges $^{L+1}I[\textbf{w}] \overset{P(t)}{\longrightarrow }~ ^{L+1}I[\textbf{z}]$, which we recognise as meta-community structure. The caution is needed because traits are not always strongly associated with species (e.g. through prokaryotic horizontal gene transfer),^5^ though they do strongly depend on the individual organism, even then they might be expressed selectively in response to the environment (the classic example being the sugar metabolism of *E. coli*).

Ecosystems of all sorts show considerable organisation of community-level processes, but the question of which level embodies supra-organism $I_f$ remains unanswered by empirical evidence. Whole communities have recovered with organisation intact following anhialation (Richards et al. 2008; Smallhorn-West et al. 2020) and community structure can survive coalescence of separate communities (Rillig et al. 2015), suggesting supra-organism embodiment. But ecological regime shifts (Scheffer et al. 2001) and community collapse (McCain et al. 2015) suggest otherwise. The explanation of physical information presented here shows its teeth by categorically asserting that unless $I_f$ is embodied as spatiotemporal pattern at some organisational level above that of the individual organism, then it is not to be found there. That leaves only metacommunity pattern and responses to the abiotic environment such as gradients in soil conditions or layers of the ocean, as sources of $^{L+1}I[\textbf{z}]$, the latter being ruled out of downward causation unless produced by niche construction processes (i.e. $I_f =~ ^{L+1}I[\textbf{w}] \overset{P(t)}{\longrightarrow }~ ^{L+1}I[\textbf{z}]$, where *P*(*t*) is niche construction and $^{L+1}I[\textbf{w}]$ is embodied no higher than $^{L}I[\textbf{z}]$).

More generally, we are left with the problem that if formal information exists to organise an ecological community, or the biochemistry of a cell, but is not to be found in physical information at the $L+1$ level it operates, then where is it? Speculatively, it might be all embodied in $L-j$ and only raised to $L+1$ when active, so that parts of it emerge functionally, but not all at the same time. We are still in need of a better explanation.

## Discussion

Physical information has been defined here as the embodiment of spatiotemporal pattern in particles of matter or fields of energy, while cybernetic information is the disembodied pattern that is subject only to the rules of logic. Distinguishing among physical, cybernetic and statistical information is not merely a pedantic point; it clarifies the way information processing by cybernetic systems, at every level of biological organisation, is animated in material systems with causal power. It also dispels confusion over the role of information in causation (e.g. Yurchenko 2023) by specifying physical cause as force-dependent, leaving cybernetic cause—logical necessity—to be strictly informational and a modern interpretation of Aristotle’s formal cause. The physical/cybernetic distinction alerts us to the difference between the model cybernetic systems (of e.g. neural or biochemical signal networks) and the physical systems they represent because the considerable amount of information used to structure them as cybernetic systems is implicit and often overlooked. Though it is obvious that we must not confuse statistical metrics of information with the information itself, the terminology (e.g. mutual information) can lead us astray. Understanding physical information as spatiotemporal pattern in force-emanating material particles enables us to identify and discriminate the information embodied at different organisational levels and this is especially useful for understanding the causal structure of organisms and the organisation of ecological communities. Explicitly considering the physical information underlying information integration (as SI or $\phi$) reveals the ‘embodied code’ that is the structural information specifying the connections among informational channels that is observed as e.g. non-zero SI. The explicit focus on physical information as the constraint of action of forces has also shown how the exclusion principle of causation does not rule against upward and downward causation, as long as forces are properly accounted for.

The value of the approach proposed here is that it brings clarity, both to understanding the underlying mechanisms of physical causation and to its limits (so we should be cautious in applying results from cybernetic analysis directly to physical systems). An example of this was shown when attempting to account for higher level structure (such as can be represented in an interaction matrix) that was not explicitly embodied as higher level spatiotemporal pattern. There remain many unanswered questions, especially concerning the level and medium of higher level information embodiment, not least in ecology and the neurological systems from which brain functions arise. The framework presented here is offered as a conceptual tool to help work those questions out.

### Philosophical comments on information

Finally, although this work does not deal primarily with the philosophical debate around concepts of information, the introduction of new concepts ($I_f$ and $I_p$) over the past few years (Farnsworth et al. 2013; Hofmeyr 2017, 2021; Farnsworth 2021, 2022, 2023, 2017) merits some comment. I started by noting, as many have before, that ‘information’ is a polysemantic concept and even qualified terms such as ‘physical information’ are not immune: that term was used by Floridi (2011) to signify the technical concepts addressed by information theory as opposed to concepts of ‘meaningful information’. That is roughly the same distinction as drawn by Krzanowski (2020), who considered physical information (labelled ‘concrete’: information$_C$) as objectively existing, devoid of meaning, quantifiable and manipulable, contrasting that with ‘abstract information’, which is subjective, semantic, perhaps semiotic, and broadly matches the everyday usage of the term information and its association with knowledge (identified here as $I_m$). Other than in the works, cited above, that deal with aspects of the organisational approach to biology (Bich and Damiano 2012; Mossio et al. 2013, 2009), the distinction between $I_f$ and $I_p$, has not been drawn; indeed $I_f$ remains unknown beyond this field of study and has so far eluded the scrutiny of philosophers. Krzanowski (2020) recognises claims in several scientific accounts (cited within) that $I_f$ and $I_p$ lumped together as information$_C$, “*expresses the organization or form of physical objects*”, though understanding of that was considered imprecise mainly due to ambiguity in the meanings of organisation and structure. To quote again “*We do not posit that information*$_C$ *is a structure in itself, because we do not know exactly what structure is, nor do we know what kind of structure would be associated with information or how this association would take place.*” (p. 7 Krzanowski 2020). Following Hofmeyr (2017, 2021) and Farnsworth (2021, 2022, 2023), we now have a precise answer, at least in the domain of classical (non-quantum, non-relativistic) physics as it may be applied to the organisation of biological systems. That answer requires that physical information is the arrangement of elementary particles (or field density) in space-time (hence physically embodied) and formal information is defined as the disembodied, abstract (some may say Platonic form) specification of the arrangement, independently of the medium of embodiment. $I_p$ is the result of instantiating $I_f$ in the physical world; conversely $I_f$ is the abstraction of mathematical pattern from observed $I_p$. To form the organisation of physical objects, elementary particles have to be arranged in a *particular* pattern and because those particles constituting objects emanate physical forces, their arrangement involves the application of physical forces, hence efficient cause—the making of things to happen in the physical world. From this, formal cause is the abstract concept of making things happen without physical forces, in a conceptual (more precisely, mathematical) domain, where the logical necessity of rules for e.g. cybernetic systems pervades. Putting this founding idea succinctly: if formal cause is efficient cause without enforcement, then formal information is physical information without embodiment.

The idea of $I_f$ and $I_p$ has already proved useful in understanding biological organisation and some of its implications (e.g. Farnsworth (2021, 2023); Farnsworth and Elwood (2023)); it provides a practical understanding of inter-level causation (and on the way, a concrete refutation of Kim ’s 1989 exclusion argument) and also explains the way network architecture may be instantiated in physical systems (Sect. 4), so it meets at least the legitimacy criterion of Lombardi et al. (2016) and also the more stringent ‘epistemic criteria’ of Anta ’s 2023—i.e. it enables scientific explanation. Furthermore, since $I_p$ is the particularising of arrangement of physical objects, it can be quantified using algorithmic information metrics (Chaitin 1966, 1990; Kolmogorov 1965) and if particulate, a limit to the information content measured is set by the number of particles to be arranged—indeed this was the original inspiration for the idea, following the quantification of molecular information in Bonchev and Trinajstić (1982). No such limit can apply in the case of $I_f$ which is an abstract, non-embodied (we might even conclude non-realist) concept. The semantic criticism of information language in science (Ch.1 Timpson (2013)) warns that we may forget that ‘information’ in communications theory is an abstract type, rather than a specific concrete object, though Anta (2023) mollifies such criticism by pointing out that scientist should be able to discern concepts of information without appreciating “the philosophicalanalytical categories of type-token”—of course most scientists will be unaware of that. The confusion of information metrics, together with their derivatives such as mutual information (collectively, * I*_*s*_), with both $I_p$ and $I_f$ and the conflation of all these with $I_m$ are more serious because in practice they have already led to misconception and faulty conclusions (some of which were addressed in Sect. 1.1). To preempt any new confusion about $I_p$ in particular, with its claim to physical foundations (e.g. Farnsworth (2022, 2023)), let us recall that it is strictly classical and does nothing to address the deeper concept of quantum information with its accompanying problems of entanglement, non-locality and Bell’s inequality (extensively dealt with in Timpson (2013)), nor causal set theory derived from efforts to reconcile those with General Relativity (Bombelli et al. 1987; Yurchenko 2023). The $I_p$ in this and the preceding works mentioned, reflects a practical engineering approach compared to the more profound physics of quantum information. Above all, the definitions of $I_p$ and $I_f$ are meant to be useful.

## Data Availability

No datasets were generated or analysed during the current study.

## Footnotes

1 The term ‘meaning’ also carries many concepts in science and its related philosophy, without general agreement or even good organisation of the subject (see e.g. Sonesson (2006)). For the present purpose, we may take meaning to be the interpretation (including representation, but not necessarily as a sign) of information. This excludes the idea of meaning as function and the subjective sense of meaning as ascribed by an external observer.

2 A precedent for this notion has not been found, but it bears scrutiny: in the absence of interpretation, nothing can be true or false, it just exists or not and true/false is elemental in a sense analogous to the binary bit, inspiring this conjecture that there is a mapping between binary logic and meaning via truth/falsehood.

3 The base level is set to 1 here in acknowledgement of the hypothesised fundamental basis of all physical causation at the Plank scale, following Bombelli et al. (1987) (and foundational to the arguments of Yurchenko (2023)), for which level zero is reserved.

4 According to General Relativity, forces act in a non-Euclidean space (curved spacetime), but for biologically relevant purposes forces do not follow curves, of e.g. of the earth’s surface, instead they cut through on a Euclidean path.

5 Grouping organisms by species is useful for population dynamic models, but species do not interact, only organisms do. Identifying traits with species is just an accounting convenience (note that prokaryotes especially show trait diversity unrelated to species (reviewed in e.g. Cohan and Koeppel (2008)) and since species cannot be the target of evolution—it is either the phenotype or genotype—, traits ought not to be strictly attached to species, though they may be associated with it).
